# Comparison of expression systems for the extracellular production of mannanase Man23 originated from *Bacillus subtilis* B23

**DOI:** 10.1186/1475-2859-12-78

**Published:** 2013-09-08

**Authors:** Haiyan Zhou, Yong Yang, Xu Nie, Wenjiao Yang, Yongyao Wu

**Affiliations:** 1 College of Bioscience and Biotechnology, Hunan Agricultural University, Changsha 410128, China

**Keywords:** Mannanase, Recombinant gene, Host bacterium, Expression system

## Abstract

**Background:**

Mannanase is an enzyme that can catalyze random hydrolysis of beta-1,4-mannosidic linkages in the main chain of mannans, glucomannans and galactomannans which are the key polymers in hemicellulose. It has been used in a number of different industrial applications including food, feed, pharmaceutical, pulp/paper industries, and second generation biofuel. To optimize the expression system of mannanase Man23 gene, two kinds of vectors and host bacteria were determined and compared.

**Results:**

Recombinants pHY-p43-*man23* and pBPS-*man23* were constructed and transferred into *Bacillus subtilis* WB600 and *Brevibacillus brevis* respectively. For mannanase Man23 gene, recombinant pHY-p43-*man23* expressed in *Brevibacillus brevis* had higher production and activity. Compared to the wild-type *Bacillus subtilis* B23, the production of recombinant pHY-p43-*man23* in *B. brevis* increased by 10 times and activity increased by 21.3%. pHY-p43-*man23* in *B. brevis* had activity at the range of 20 ~ 70°C but its optimum temperature was 50°C and had activity from pH 4 ~ 10 but its optimum pH was around 7. This demonstrated the recombinant had improved stability as well.

**Conclusions:**

Mannanase is an important industrial enzyme and combination of vector pHY-p43 and host *Brevibacillus brevis* is a novel expression system for a mannanase decoding gene. This work aims at exploring a better expression system of mannanase Man23 decoding gene for industrial application.

## Background

β-Mannanase, an extracellular enzyme, has hemicellulase activity or the activities of both hemicellulase and cellulase [1]. Mannanase can catalyze random hydrolysis of beta-1,4-mannosidic linkages in the main chain of mannans, glucomannans and galactomannans which are the key polymers in hemicellulose [2]. It is widely used by industries including food processing, feed, oil mining, paper making, pharmaceutical, and second generation biofuel [3,4]. It is especially involved in breaking down plant tissues by degrading mannan polymers in the cell walls [5].

Mannanases once were isolated from plants [6], marine mollusk [7], and a body of bacteria and fungi [8,9]. According to the hydrophobic cluster analysis of reported mannanases, they have been classified into glycoside hydrolase (GH) families 5 and 26. In this paper, mannanase Man23 which originated from *Bacillus subtilis* B23 belongs to family 26.

Wild-type mannanase Man23 has high activity and stability. We found that the sites of H129, E159, H190, E191, W196, F197, W198, and W199 on mannanase Man23 are relevant to the activity and substrate binding. The mutations at the sites of H129, H190, and W198 increased activity by 3.5-, 2.2-, and 3.8-fold, respectively (Haiyan Z, Xu N, Yong Y, Wenjiao Y, Yongyao W: Engineering mannanase Man23 decoding gene from *Bacillus Subtilis* B23 by semi-rational design, submitted). Herein, in order to promote the production and stability for meeting its commercial usage, our team had a further study to optimize the expression system of mannanase Man23. We evaluated two vectors, pHY-p43 and pBKE50, and two host bacteria, *Bacillus subtilis* (*B. subtilis*) WB600 and *Brevibacillus brevis* (*B. brevis*), to figure out how the recombinant expression plasmids performed in the two novel hosts. This work provides insights into how an optimized expression system worked on mannanase Man23 gene and moreover, it throws a clue to select a more suitable expression system and a host bacterium for cellulase or hemicellulase gene.

## Results

### Comparison of gene *man23* expressed in host *B. subtilis* WB600 and *B. brevis*

For the economical reason, it is necessary to construct the stable expression systems for producing recombinant proteins. At present, about 60% of the commercially available enzymes are produced by *Bacillus* species [10]. In comparison of *Escherichia coli* (*E. coli*), *B. subtilis* is an attractive host for enzyme production since it is nonpathogenic and has a high capacity of secreting extracellular proteins directly into the medium [11]. However, *B. subtilis* greatly reduces the production and intactness of the secreted proteins because of it secrets at least seven extracellular proteases [12]. A series of extracellular-protease-deficient *Bacillus* strains were improved as a cell factory for secreting target proteins.

*B. subtilis* WB600 is a six-extracellular-protease-deficient strain and it was shown that in this strain, protein degradation is minimized and the production of some proteins was improved compared to that in the wild-type [13].

*B. brevis* is a naturally extracellular-protease-deficient strain and has been known to exhibit high productivity of heterologous proteins into the culture medium but produce little extracellular protease [14,15], which enables a high recovery of the target proteins with minimal degradation. Thus it has been used as a good host for production of heterologous proteins. Importantly, these two hosts have the clearly-known metabolic background.

**Figure 1:**
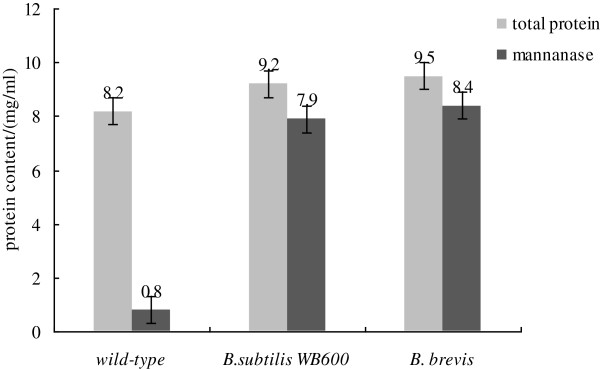
**The production of total proteins and mannanase expressed by pHY-p43-*man23 *in different hosts.**

The gene *man23* using pHY-p43 as the expression vector firstly was introduced into the two hosts *B. subtilis* WB600 and *B. brevis*. The production yield of total proteins and mannanase Man23 in different hosts was shown in Figure 1. In comparison to the wild-type, *B. bacillus* WB600 and *B. brevis* displayed a better ability to express gene *man23*. The production of total proteins in *B. bacillus* WB600 and *B. brevis* increased by 12% and 16%, meanwhile, the production of mannanase Man23 increased by 9.8-fold and 10.5-fold and mannanase activity increased by 21% and 21.3% respectively.

**Figure 2:**
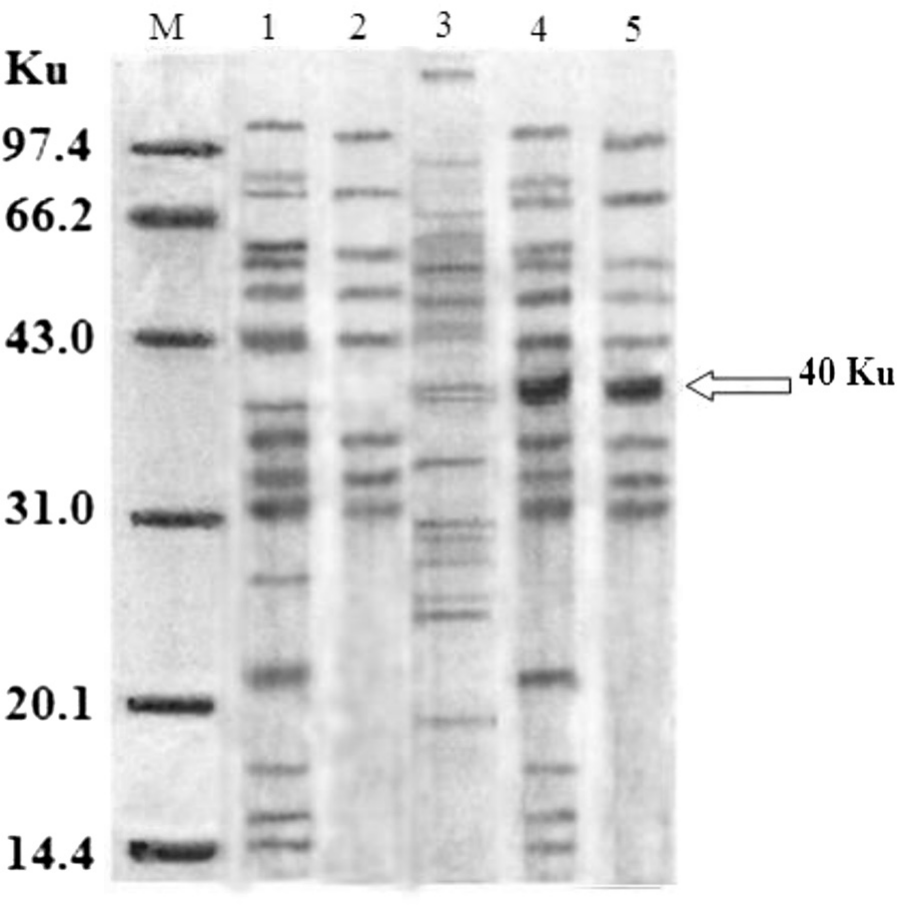
**SDS-PAGE of gene *man23 *expression products in different hosts.** M: standard protein molecular weight marker; 1: crude extract of *B. bacillus* WB600; 2: crude extract of *B. brevis*; 3: crude extract of wild-type host; 4: crude extract of *B. bacillus* WB600 (with pHY-p43-*man23*); 5: crude extract of *B. brevis* (with pHY-p43-*man23*). The target band arrow pointed to is mannanase band.

SDS-PAGE was used to identify the secreted protein from different hosts and the result was shown in Figure 2. From the diagram, *B. subtilis* WB600 and *B. brevis* displayed their excellent and high-efficient production for mannanase Man23. Especially, *B. brevis* had a clearer electrophoresis background compared to WB600 and wild-type host, which suggested *B. brevis* produced more mannanase Man23 and less other proteins. Thus, the purity of mannanase Man23 from *B. brevis* was highest among the tested hosts. In comparison with *B. subtilis* WB600 and *B. brevis*, the total protein production in *B. brevis* was higher than *B. subtilis* WB600 by 3% and mannanase Man23 production was higher by 6.3%. Table 1 demonstrated the mannanase activity produced in *B. brevis* was higher than *B. subtilis* WB600 by 0.6%.

**Table 1:** **Comparison of mannanase activity produced by pHY-p43-*man23 *in different hosts**

**Host bacterium**	**Mannanase activity(U/mg)**
Wild-type	177.43 ± 0.5
*B. bacillus* WB600	214 ± 0.3
*B. brevis*	215.3 ± 0.5

Considering the data above and mannanase purity, *B. brevis* has been used as the host for expressing gene *man23* in our following work.

### Comparison of the expression vectors pHY-p43 and pBKE50 for gene *man23*

Plasmid pBKE50 is a shuttle vector which includes the replication origin of pUB110 and the erythromycin-resistance gene of pGK12. Plasmid pHY-p43 is also a shuttle vector which includes the strong promoter P43. In our work, these two vectors were introduced to express gene *man23* in *B. brevis*.

Mannanase Man23 encoding gene was identified to be 1100 bp and was registered on Genbank [16]. Gene *man23* was cloned using primer P1 and P2 to add *BamH* I and *EcoR* I restriction sites for the link with vector pHY-p43 and using primer P3 and P4 to add *BamH* I and *Kpn* I restriction sites for the link with vector pBKE50.

Both recombinants, pHY-p43-*man23* and pBPS-*man23*, had excellent performance when they transformed into the host *B. brevis* and the results about production yield and mannanase activity of these two recombinants were shown in Table 2. Apparently, both recombinants produced much more mannanase and higher activity. The amount of total proteins expressed by pHY-p43-*man23* and pBPS-*man23* was enhanced by 19% and 4% compared to the wild-type. Moreover, comparing to the wild-type, the amount of mannanase Man23 increased by 11.25-fold and 8.05-fold and the mannanase activity increased by 18% and 3.4% respectively. From the data, it can be easily figured out that pHY-p43-*man23* had more proteins production by 14.45%, more mannanase production by nearly 40% and higher mannanase activity by 14% than pBPS-*man23*. For this reason, in our following work, pHY-p43 has been used as the vector to express gene *man23*.

**Table 2:** Comparison of the production yield and mannanase activity produced by different expression plasmids

**Expression plasmid**	**The amount of total protein(mg/ml)**	**The amount of mannanase Man23(mg/ml)**	**Mannanase activity(U/mg)**
Wild-type	8.0 ± 0.06	0.72 ± 0.02	188.5 ± 0.3
pHY-p43-*man23*	9.5 ± 0.05	8.1 ± 0.05	222.5 ± 0.2
pBPS-*man23*	8.3 ± 0.05	5.8 ± 0.03	195 ± 0.2

### Biochemical characteristics of recombinant mannanase Man23 expressed by pHY-p43-*man23* in host *B. brevis*

**Figure 3:**
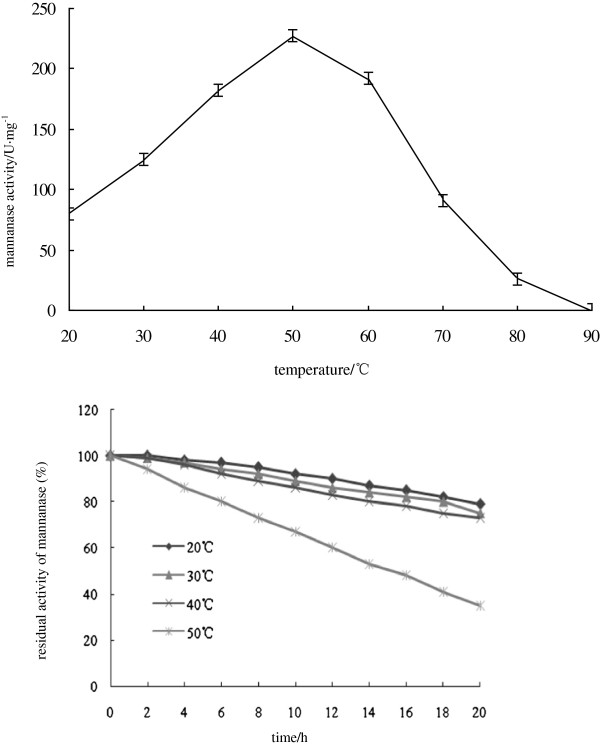
Effects of temperature on activity and stability of recombinant mannanase Man23.

To obtain the details about recombinant mannanase Man23, herein we determined its biochemical characteristics. It had activity at the temperature range of 20 ~ 70°C and its optimum temperature is 50°C (Figure 3). When the recombinant Man23 had been maintained at different temperature for 20 hours, it displayed high stability at temperature of 20 ~ 40°C and reduced sharply at 50°C in 20 hours (Figure 3). Compared to the wild-type which had the optimum temperature 45°C and the activity range of 30 ~ 60°C, the recombinant mannanase Man23 displayed a broader temperature range with high activity and a better heat tolerance.

**Figure 4:**
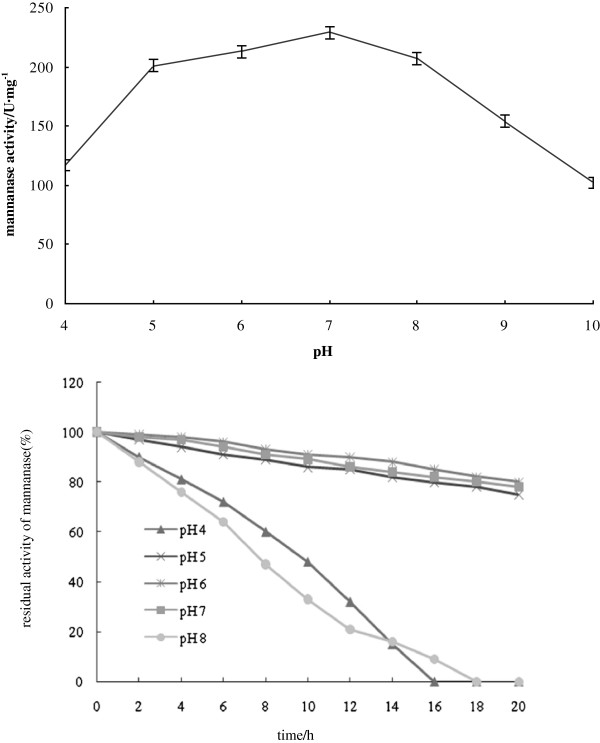
Effects of pH on activity and stability of recombinant mannanase Man23.

Meanwhile, the optimum pH of wild-type was 6.5 and it had activity in the pH range of 4 ~ 9. Nevertheless, recombinant mannanase Man23 had activity in a broader pH range of 4 ~ 10 and its optimum pH shifted slightly to around pH 7 (Figure 4). When being reserved in the phosphate buffer of pH 5 ~ 7 for 20 hours, the recombinant Man23 still kept 90% of activity but when out of pH 5 ~ 7 it was not very stable (Figure 4).

The kinetic parameters of recombinant Man23 degrading locust bean gum were determined in 50 mmol/L phosphate buffer of pH 7.0 at 50°C. Through the Lineweaver-Burk Plot, the values of *K*_*m*_ and *V*_*max*_ were 0.38 mg · ml^-1^ and 301 μmol · mg^-1^ · min^-1^.

## Discussion

Endo-1,4-β-Mannanase has been classified into two glycosyl hydrolase families, GH family 5 and 26, according to the amino acid sequence similarities and hydrophobic cluster [17]. From the sequence analysis, mannanase Man23 mentioned in this paper belongs to family GH26.

There have been some reports about the optimization of expression system and the characterization of recombinant mannanase as summarized in Table 3. The optimal pH, temperature and the stability of mannanases reported were varying depending on the sources. Some mannanases screened by Katrolia P. [18] and Songsiriritthigul C. [2] had similar enzyme characteristics with mannanase Man23 and mannanase screened by Vu TT [19] had similar optimum pH with mannanase Man23 but the latter had broader pH activity range and higher thermo-stability. The activity and kinetic parameters of the mannanases from different sources varied greatly mainly because there are lots of differences with the structure of these mannanases. Usually, some thermostable mannanases tend to have lower specific activity in comparison with their mesophilic counterparts [20]. When comparing to other mannanases, it worth to be noticed that different substrates and individual techniques whittled down the accuracy for evaluating mannanase activity.

**Table 3:** Properties of various mannanase recombinants

**Organism**	**Family GH**	**Expression vector or plasmid**	**Properties of recombinant mannanase**	**Ref**
**(wild-type/expression host)**				
*Stearothermophilus*/ *E. coli*		Plasmid pH6EX3	The recombinant had thermostability similar to the native enzyme; The values of V_*max*_ and K_*m*_ were 384 U/mg and 2.4 mg/ml.	[21]
*Bacillus circulans* CGMCC 1416/ *E. coli*	GH5	pET-22b(+)	The activity was 481.55 U/mg, the optimal temperature was 58°C and pH was 7.6.	[22]
*B. subtilis* Bs5/ *E. coli*			The optimal temperature was 35°C, the optimal pH was 5.0 and pH range was wide from 3.0-8.0.	[23]
*Bacillus* sp. JAMB-750/ *B. subtilis*	GH26		The optimal pH was around pH 10.	[24]
*B. subtilis* WL-3/*B. subtilis* 168	GH26	Vector pJ27Δ88U	The mannanase activity reached a maximum level of 450 U/ml.	[25]
*Aspergillus sulphureus*/ *P. pastoris*	GH5		The highest activity was at pH 2.4 and 50°C; pH range was 2.2-8.0; it was stable below 40°C. The K_*m*_ and V_*max*_ values for locust bean gum at 50°C and pH 2.4 were 0.93 mg/mL and 344.83U/mg, respectively.	[26]
*Bispora* sp. MEY-1/ *P. pastoris*	GH5		The recombinant was acidophilic with highest activity at pH 1.0-1.5; the optimal temperature was 65°C; The specific activity, K_*m*_, and V_*max*_ for locust bean gum was 3,373 U/mg, 1.56 mg/ml, and 6,587.6 mmol/min/mg, respectively.	[27]
*B. subtilis* strain G1/ *P. pastoris* GS115			The recombinant had an optimum temperature of 45°C and optimum pH of 6.5; the enzyme was stable at temperatures up to 50°C (for 8 h) and in the pH range of 5–9.	[19]
*Humicola insolens* Y1/ *P. pastoris*	GH5		The recombinant had a specific activity of 1,122 U/mg and exhibited optimal activity at pH 5.5 and 70°C; it had excellent pH stability at pH 5.0-12.0 and was highly thermostable at 50°C.	[28]
*Penicillium freii* F63/ *P. pastoris*	GH5		The recombinant was optimal at pH 4.5 and 60°C and exhibited good stability over a broad pH range from acidic to alkaline (>85.0% activity at pH 4.0-9.0, >70.0% activity at pH 10.0 and 43.7% even at pH 12.0).	[29]
*B. subtilis* MAFIC-S11/ *P. pastoris*			The expression level was improved by 2-fold; the recombinant enzyme showed its highest activity of 24,600 U/ml after 144-h fermentation; the optimal temperature and pH were 50°C and 6.0, respectively; the specific activity was 3,706 U/mg; the kinetic parameters V_*max*_ and K_*m*_ were determined as 20,000 U/mg and 8 mg/mL, respectively.	[30]
*Aspergillus aculeatus*/ *Aspergillus oryzae*		Vector pYES2.0	The pH optimum was pH 5.0 and a temperature optimum was 60-70°C.	[31]
*Bacillus* sp. N16-5/ *Kluyveromyces cicerisporus*			The maximum yield of recombinant reached 3,795 U/ml; it exhibited similar pH optima, temperature optima, and substrate specificities to its wild-type; its stability was about 7% higher than that of wild-type from pH 9–11 and had about 10% higher stability than wild-type from 60°C to 80°C.	[32]

Δ: delta, Vector pJ27Δ88U is a plasmid with a strong *B. subtilis* promoter.

From the references, mannanase encoding gene from different organisms was transformed to express in prokaryotic cells such as *E. coli* and *B. subtilis* and eukaryotic cells such as yeasts. Mannanase Man23 encoding gene was hardly secreted from *E. coli*, therefore *E. coli* would not be a good option if the secretary proteins were expected. Moreover, the secretion of proteins would simplify the extraction procedure. Both *B. subtilis* WB600 and *B. brevis* are effective hosts for secretary proteins since there are abundant of vectors and regulatory elements for *Bacillus* species to help proteins folding [33,34]. *B. brevis* especially has its advantages to be the expression host for gene *man23* because *B. brevis* can secrete disulfide-bond-promoting factors to help proteins folding [35]. In our previous work, mannanase Man23 was found one disulfide bond formed between Cys90 and Cys110. Actually, our results about two tested hosts demonstrate *B. brevis* is more suitable for gene *man23* indeed. Eukaryotic cells usually have the capacity to express extracellular proteins efficiently. However, considering the economic reasons, prokaryotic cells have their advantages because of the shorter production period and the simpler extraction process compared to that of eukaryotic cells.

Each expression host has one or more applicative vectors. Some reported mannanase genes used their native expression vector, and while some were recombined into other vectors. Vector pJ27Δ88U was tested and suitable for use in *B. subtilis* 168. In our work, plasmid pBKE50 and pHY-p43 are the shuttle vectors of *E. coli* and *Bacillus*. For gene *man23*, the promoter p43 on the vector pHY-p43 showed high efficiency to manipulate mannanase Man23 encoding gene in *B. brevis*. It deserves the further researches on other applicable vectors for gene *man23* expression in *B. brevis*.

## Conclusions

The system composed of vector pHY-p43 and host *B. brevis* is a novel expression system for mannanase encoding gene. Our results demonstrate that this novel system is efficient for expressing and secreting mannanase Man23 encoding gene, which deserves to be considered for indus-trial applications. Additionally, this expression system could be adopted for producing other enzymes as well.

## Methods

### Extraction of genomic DNA and plasmid DNA

Genomic DNA was extracted by SDS method [36] and plasmid DNA was extracted by alkaline lysis method [37].

### Cloning of mannanase Man23 encoding gene

Through the sequence alignment with high homology, two couples of primers were designed to clone mannanase Man23 encoding gene from the whole DNA of wild-type. One couple used to clone the decoding gene linked with vector pHY-p43 is P1:5′-CGCGGATCCATGCCTACTAAGT-3′ (underline is *BamH* I restriction site) and P2:5′-CGGAATTCTGATTCAGCTATCTGTG-3′ (underline is *EcoR* I restriction site). The other couple used to clone the decoding gene linked with vector pBKE50 is P3:5′- CGCGGATCC ATGCCTACTA AGT-3′ (underline is *BamH* I restriction site)and P4:5′-GGGGTACCTGATTCAGCT ATCTGTG-3′ (underline is *Kpn* I restriction site).

The PCR reaction is 94°C for 5 min, followed by 30 cycles of 94°C for 30 s; 56°C for 30 s; 72°C for 1 min, then 72°C for 10 min, 4°C thereafter. Cloning products were detected and recycled according to reference [38].

### Construction of expression plasmid pHY-p43-*man23*

**Figure 5:**
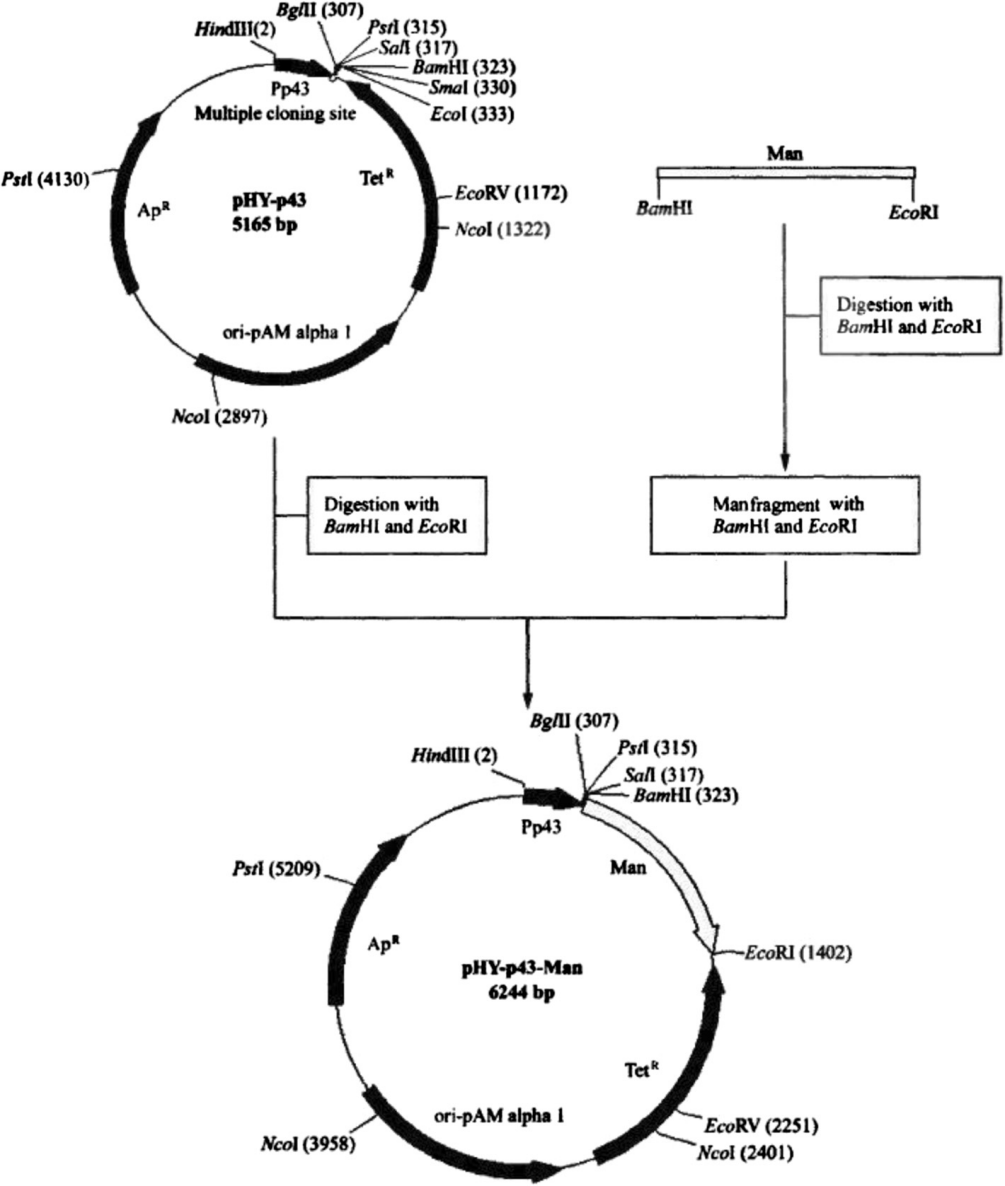
**The construction of expression plasmid pHY-p43-*man23.***

Plasmid pHY-p43-*man23* was constructed from plasmid pHY-p43 and gene *man23* with *BamH* I and *EcoR* I restriction sites. The plasmid pHY-p43 (CICIM MMB0050) was purchased from CICIM-CU. The procedure of construction was represented in Figure 5.

### Construction of expression plasmid pBPS-*man23*

**Figure 6:**
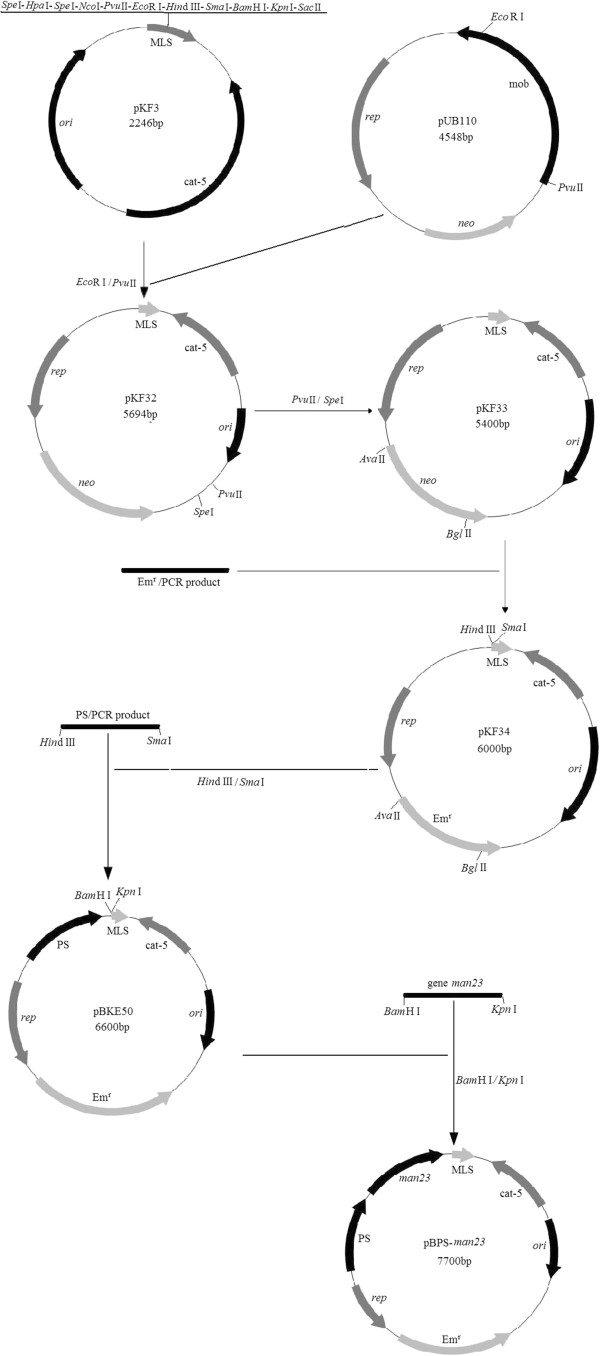
**The construction of expression plasmid pBPS-*man23.*** MLS: multiple clone sites; cat-5: chloramphenicol resistance gene; Em^r^: erythromycin resistance gene; *ori*: replication of *E. coli*; *rep*: replication of *B. brevis*; PS: decoding gene of the promoter and signal peptide of *B. brevis.*

Plasmid pBPS-*man23* was constructed from plasmid pBKE50 and gene *man23* with *BamH* I and *Kpn* I restriction sites. Plasmid pBKE50 was constructed basing on initial plasmids pKF3 [39] and pUB110 [40]. After digestion and re-link, the intermediate plasmid pKF33 was linked with erythromycin-resistance gene (Em^r^) to form plasmid pKF34. Another couple of primers, P5 and P6, were designed to clone the decoding gene of the promoter and signal peptide of *B. brevis*. P5 is 5′-CCCAAGCTTCGTGAG AATGCGTACCAAA-3′ (underline is *Hind III* restriction site) and P6 is 5′-TCCCCCGGGCGAAAGCCATGGGAGCAAC-3′ (underline is *Sma I* restriction site). The PCR reaction was 94°C for 5 min, followed by 30 cycles of 94°C for 40 s; 60°C for 30 s; 72°C for 50 s, then 72°C for 5 min, 4°C thereafter. The decoding gene of the promoter and signal peptide (PS) of *B. brevis* and plasmid pKF34 were digested with *Hind III* and *Sma I* and then linked together to form the shuttle vector pBKE50. The details of construction procedure were represented in Figure 6.

### Transformation and expression of the recombinant plasmids

*B. bacillus* WB600 and *B. brevis* are both the *Bacillus* species hosts, therefore the approaches recombinant plasmids transforming into the hosts are similar and elaborated as following.

The host cells were shaking cultured in T2 medium [41] overnight at 37°C and then cultures were refreshed into 5 ml of T2 medium by 1% of incubation amount and continued culturing till the late stage of logarithmic growth. The host cells were collected and suspended in 5 ml of 50 mmol/L Tris–HCl (pH 7.5) for a while and then cultured in 5 ml of 50 mmol/L Tris–HCl (pH 8.5) at 37°C for one hour. The host cells were collected into 0.5 ml of TP medium (phosphate buffer (1.905 g KH_2_PO_4_, 0.852 g Na_2_HPO_4_, 100 ml) mixed with 2 × T2 medium in proportion of 1:1) and at the same time, 100 μl of TE/TP buffer (TE buffer(10 mmo1/L Tris–HCl, pH 7.5 and l mmo1/L EDTA) mix with TP medium in proportion of 1:1) blended with 20 ul recombinant plasmids of 10 ng/ul and 1.5 ml PEG solution (PEG6000 400 g, 500 ml phosphate buffer). The mixture was incubated at 37°C for 10 min and then the host cells were recollected into 1 ml MT medium (MgCl_2_ in T2 medium with final concentrate of 20 mmol/L) and agitated at 37°C for 30 min. After antibiotics Amp of 50 ug/ml and Tet of 40 ug/ml were added into MT medium, the cultivation was continued for another 2 h. The plate cultures with 100 ug/ml of Amp and 60 ug/ml of Tet were used successively to screen individual colonies.

### Isolation of native and recombinant mannanase Man23

Crude proteins were prepared from supernatant after centrifuge and then successively purified through salting-out, molecular sieve chromatography Sephadex G-100 and ion-exchange column chromatography. The isolation procedure was carried out according to reference [42].

### Determination of recombinant mannanase activity and biochemical characteristics

Protein concentration was measured using the Bradford assay [6]. Mannanase activity assay was improved from the method of reducing sugar assay [43,44]. One unit of enzyme activity was defined as the amount of enzyme liberating 1 μmol mannose per minute at 50°C and pH 6.8. The activity formula was as follows:

Mannanase activity(U/ml) = 5.56C_e_V_de_/V_je_V_s_t

5.56 the mole value of 1.0 mg mannose, μmol

C_e_ amount of mannose produced from hydrolysis, mg

V_de_ metered volume of enzyme solution, ml

V_je_ volume of enzyme solution added into the reaction mixture, ml

V_s_ volume of substrate solution, ml

t time, min

Specific activity of mannanase (units per milligram) = 5.56C_e_V_de_^2^/C_p_V_je_V_s_t

C_p_ amount of total proteins, mg

The optimal temperature of mannanase Man23 was evaluated by the activity assay at 20, 30, 40, 50, 60, 70, 80, 90°C for 10 min. The assay was performed with 1 ml mannanase solution (~1.0 mg/ml) at pH 6.8 and 50 mmol/L phosphate buffer.

The optimal pH was evaluated by the activity assay in different pH values of 50 mmol/L phosphate buffer for 10 min and temperature was maintained at 50°C. The mannanase amount was same as above.

Thermostability of mannanase Man23 was measured by incubating the samples for 20 hours at different temperatures and then plotting the residual activity versus the incubation time. The pH tolerance was measured by incubating the samples for 20 hours at different pH and then plotting the residual activity versus the incubation time.

### Electrophoresis analysis of protein samples

The proteins extracted from wild-type, *B. bacillus* WB600 and *B. brevis* were analyzed by SDS-PAGE [45]. Marker was purchased from TIANGEN biotech co., LTD and it includes six bands of 97.4, 66.2, 43.0, 31.0, 20.1 and 14.4 Ku.

### Statistical analysis

Data were presented as the mean ± standard error of the mean. Results were compared with the analysis of variance and Fisher’s protected least-significant difference tests, with a significance of *P* < 0.05.

## Competing interests

The authors declare that they have no competing interests.

## Authors’ contributions

HZ conceived and designed the study, carried out the molecular genetic studies, and drafted the manuscript. YY and XN carried out the genetic studies and the determinations. WY participated in determinations and performed the statistical analysis. YW conceived of the study, participated in its design and helped to draft the manuscript. HZ, YY, XN, WY and YW reviewed and edited the manuscript. All authors read and approved the final manuscript.
